# Supplementary material for: Helminth Parasites among Rodents in the Middle East Countries: A Systematic Review and Meta-Analysis
Source: Animals (Basel). 2020 Dec 9;10(12):2342. doi: 10.3390/ani10122342 (PMC7764038; doi:10.3390/ani10122342)
Supplement: Supplementary file 1 [file animals-10-02342-s001.zip › Supplimentary file/Supplimentary Table S2.docx]

**Table S2**. Extracted data from the selected 65 studies.

| **Sl No.** | **Author and Reference** | **Country and Location** | **Season and year of sampling** | **Rodent details** | **Rodent Helminths** | **Associating factors** | **Remarks** |
| --- | --- | --- | --- | --- | --- | --- | --- |
| 1 | Abd el-Wahed *et al.* [1] | Egypt, Qalyobia governorate | - | *Rattus norvegicus* (N=108)  *Rattus rattus alexandrines* (N=64)  n=172, Rc=48, Rn=12, Ra=14 | **Cestode**  *Cysticercus fasciolaris*  *Hymenolepis diminuta*  **Nematode**  *Strongyloides* spp. |  |  |
| 2 | Abdel-Gaber *et al.* [2] | Egypt |  | Mus musculus  n=50, R=28, Rn=28 | **Nematode**  *Aspiculuris tetraptera* |  | Spelling corrected from *Aspicularis tetrptera* to *Aspiculuris tetraptera* |
| 3 | Abdel-Gaber [3] | Egypt, Cairo University, Cairo | March 2014 to February 2015 | *Mus musculus* (F=30, M=30)  n=60, R=45, Rn=45 | **Nematode**  *Syphacia obvelata* | The prevalence of *Syphacia obvelata* infection was higher in male mice than female mice. |  |
| 4 | Abdel-Salam *et al.* [4] | Egypt, sohag governorate | - | *Arvicanthis niloticus* (N=40)  *Mus musculus* (N=40)  *Rattus rattus alexandrines* (N=40)  *Rattus rattus frugivorous* (N=40)  n=160, R=87, Rc=45, Rn=42 | **Cestode**  *Hymenolepis diminuta*  *Hymenolepis nana*  *Raillietina* sp.  **Nematode**  *Syphacia obvelata*  *Trichocephalus muris* | Cestode are more prevalent than nematodes. |  |
| 5 | Abou-Zinadah and Fouad [5] | Saudi Arabia, Jiddah | - | *Acomys dimidiatus* (N=32)  *Jaculus jaculus* (N=10)  *Meriones crassus* (N=34)  *Rattus norvegicus* (N=18)  *Rattus rattus* (N=49)  n=143, R=21, Rt=21 | **Trematode**  *Fasciola* spp. | The snail *Dircooelium dendriticum* may the cause of the spread of the trematode *Fasciola* in Saudi Arabia. |  |
| 6 | Abu-Madi *et al.* [6] | Qatar, Doha | February 2002 to April 2003 | *Rattus norvegicus* (F=96, M=83)  n=179, R=64, Rc=64 | **Cestode**  *Hymenolepis diminuta* | The abundance of *Hymenolepis diminuta* can be increased due to increased rat population and age. The rodent host sex does not have any effect on the cestode abundance. Prevalence and abundance of H. diminuta is corelated with flea *Xenopsylla astia.* |  |
| 7 | Abu-Madi *et al.* [7] | Qatar, Doha | June, 1998- April, 1999 | *Rattus norvegicus* (F=84, M=52)  n=136, R=24, Rc=24 | **Cestode**  *Hymenolepis diminuta* | Adult rats are more prevalent with *Hymenolepis diminuta* than youngs. *Hymenolepis diminuta* has explicit sex or seasonal pattern of prevalence on rat as well as presence of *X. astia* |  |
| 8 | Al Hindi and Abu-Haddaf [8] | Palestine, Gaza | January 2008-May, 2009 | *Rattus rattus* (F=15, M=26)  n=41, R=25, Rc=15, Rn=10 | **Cestode**  *Hymenolepis diminuta*  **Nematode**  *Heligmonoides josephi*  *Strongyloides* spp.  *Syphacia obvelata* | The prevalence of gastrointestinal parasites among male rats are more than female rats. |  |
| 9 | Allymehr *et al.* [9] | Iran, Northwest Iran | April-December, 2010 | *Mus musculus* (F=52, M=25)  n=77, R=43 | **Cestode**  *Cysticercus fasciolaris*  *Hymenolepis diminuta*  **Nematode**  *Aspiculuris tetraptera*  *Syphacia muris*  *Syphacia obvelata* | House mouse is a significant pest in poultry facilities and harbors vectors and reservoirs of poultry and zoonotic diseases. |  |
| 10 | Al-Quraishy *et al.* [10] | Saudi Arabia, Riyadh | April to May 2012 | *Meriones libycus*  n=40, R=18, Rc=18 | **Cestode**  *Hymenolepis diminuta* |  |  |
| 11 | Al-Qureishy [11] | Saudi Arabia, Riyadh: Industrial region and Al-  Karj road | October 2007 - March 2008 | Meriones libycus syrius  n=105, R=72, Rc=72 | **Cestode**  *Hymenolepis diminuta* |  |  |
| 12 | Antoniou *et al.* [12] | Cyprus | 2000-2003 | *Mus musculus* (N=3)  *Rattus norvegicus* (N=402)  *Rattus rattus frugivorous* (N=220)  n=625, R=92, Rc=92, Rn=1 | **Cestode**  *Cysticercus fasciolaris*  *Hymenolepis diminuta*  **Nematode**  *Physaloptera* spp. | Rodent abundance can vary with rodent collection site. Season of rat collection, rat body weight, and sex does not affect helminth prevalence | Spelling corrected from *Physallopteara* to *Physaloptera* |
| 13 | Arzamani *et al.* [13] | Iran, North Khorasan Province, Northeast Iran | 2011-2013 | *Allactaga elater* (N=13)  *Apodemus witherbyi* (N=7)  *Calomyscus elburzensis* (N=4)  *Calomyscus mystax* (N=3)  *Cricetulus migratorius* (N=2)  *Dryomys nitedula* (N=4)  *Ellobius fuscocapillus* (N=1)  *Meriones libycus* (N=13)  *Meriones persicus* (N=24)  *Microtus paradoxus* (N=3)  *Mus musculus* (N=10)  *Nesokia indica* (N=18)  *Rhombomys opimus* (N=11)  n=113, R=58 | **Cestode**  *Cysticercus fasciolaris*  *Hymenolepis diminuta*  *Skrjabinotaenia lobata*  **Nematode**  *Aspiculuris tetraptera*  *Nippostrongylus brasiliensis*  *Protospirura seurat*  *Rictularia ratti*  *Streptopharagus kuntzi*  *Syphacia obvelata*  *Trichuris muris*  *Trichuris* spp. | *Syphacia obvelata* are the most frequent nematodes in rodents in Iran. | Spelling corrected from *skrjabinitaenia* to *skrjabinotaenia*; *Rictolaria* to *Rictularia* |
| 14 | Ashour and Lewis [14] | Egypt | - | *Gerbillus gerbillus* (N=128)  *Mus musculus* (N=104)  n=232 | **Nematode**  *Gongylonema aegypti* |  |  |
| 15 | Avcioglu *et al.* [15] | Turkey, Erzurum, Eastern Anatolia | February-December, 2016 | *Apodemus* spp. (N=93)  *Mesocricetus* spp. (N=12)  *Microtus* spp. (N=391)  n=496, R=5, Rc=5 | **Cestode**  *Echinococcus multilocularis* |  |  |
| 16 | Azzam *et al.* [16] | Egypt, Giza governorate | - | *Acomys cahirinus* (N=2)  *Arvicanthis niloticus* (N=13)  *Jaculus jaculus* (N=1)  *Mus domesticus* (N=3)  *Mus musculus* (N=4)  *Rattus norvegicus* (N=6)  *Rattus rattus alexandrinus* (N=12)  *Rattus rattus frugivorous* (N=24)  *Rattus rattus rattus* (N=2)  n=77, R=20, Rt=20 | **Trematode**  *Echinostoma liei* |  |  |
| 17 | Azzam *et al.* [17] | Egypt, Giza and Ismailia governorate | 2013-2015 | *Acomys cahirinus* (N=2)  *Arvicanthis niloticus* (N=18)  *Jaculus jaculus* (N=1)  *Mesocricetus auratus* (N=3)  *Mus domesticus* (N=3)  *Mus musculus* (N=4)  *Rattus norvegicus* (N=6)  *Rattus rattus alexandrines* (N=15)  *Rattus rattus frugivorous* (N=30)  *Rattus rattus rattus* (N=2)  *n=84, R=47, Rc=34, Rt=31, Rn=10* | **Trematodes**  *Echinostoma liei*  *Schistosoma mansoni*  *Fasciola hepatica*  **Nematodes**  *Angiostrongylus cantoniensis, Trichuris sp.*  *Enterobius vermicularis*  **Cestode**  *Hymenolepis nana* |  |  |
| 18 | Behnke *et al.* [18] | Egypt, St Katherine, Central Sinai | May-June, 1997 | *Acomys cahirinus dimidiatus* (F=33, M=28)  n=61 | **Cestode**  *Rodentolepis negevi*  **Nematode**  *Aspiculuris africana*  *Dentostomella kuntzi*  *Mastophorus muris*  *Protospirura muricola*  *Syphacia minuta* | There is no effect of parasite abundance on rodent sex. There is a significant effect of the site of rodent collection or the rodent age on nematode burden. |  |
| 19 | Behnke *et al.* [19] | Egypt, St Katherine, Central Sinai | August to September 2000 | *Acomys dimidiatus* (F=89, M=79)  n=168 | **Cestode**  *Mesocestoides* sp.  *Jayeuxiella rossicum*  *Hymenolepis nana*  *Rodentolepis negevi*  *Witenbergitaenia* sp.  **Nematode**  *Aspiculuris africana*  *Dentostomella kuntzi*  *Mastophorus muris*  *Protospirura muricola*  *Streptopharagus kuntzi*  *Streptopharagus numidicus*  *Syphacia minuta* | *Spiruride* nematode burden varies significantly with rodent collection sites. *Oxyuroid* nematodes do not vary with rodent collection sites or rodent sex, age, or abundance. | *Hymenolepis nana* is the modern name of *Rodentolepis nana* |
| 20 | Beiromvand *et al.* [20] | Iran, Razavi Khorasan Province, Northeast Iran | October 2010-July, 2011 | *Apodemus witherbyi* (N=9)  *Microtus transcaspicus* (N=54)  *Mus musculus* (N=15)  *Nesokia indica* (N=1)  n=79, R=28, Rc=28 | **Cestode**  *Echinococcus multilocularis*  *Taenia* spp. |  |  |
| 21 | Borji *et al.*  [21] | Iran, Razavi Khorasan Province, Northeast Iran | October 2011 to August 2012 | *Mesocricetus auratus* (F=51, M=49) n=100, R=36, Rc=36 | **Nematode**  *Trichurata (undetermined)*  *Syphacia* sp. | There is no significant difference between age, sex, litter, breeding place, breeding style, and anthelmintic infection with individual helminth infection in Iranian hamsters. |  |
| 22 | Celebi *et al.* [22] | Turkey, Giresun privince | - | *Apodemus flavicollis*  n=1, R=1, Rn=1 | **Nematode**  *Capillaria hepatica* |  |  |
| 23 | Ebrahimi *et al.* [23] | Iran, Azarbaijan Province, North-west Iran | June 2012- August 2015 | *Mus musculus* (F=98, M=58)  n=156, Rc=20, Rn=23 | **Cestode**  *Hymenolepis diminuta*  *Hymenolepis nana*  **Nematode**  Aspiculuris tetraptera  Syphacia muris  Syphacia obvelata | Males are more prevalent than a female with the gastrointestinal parasite. | Spelling corrected from *Aspicularis* to *Aspiculuris* |
| 24 | el Gindy *et al.* [24] | Egypt, Suez Governorate | - | *Arvicanthis niloticus*  *Rattus norvegicus*  *Rattus rattus* | **Cestode**  *Andrya neotomae*  *Hymenolepis diminuta*  *Cysticercus fasciolaris*  *Hymenolepis nana*  **Nematode**  *Aspiculuris tetraptera*  *Syphacia obvelata*  *Syphacia muris*  *Protospirura muricola*  **Trematode**  *Echinostoma callawayensis*  *Schistosoma mansoni* |  | Spelling corrected from *tetrapetra* to *tetraptera*.  *Cysticercus fasciolaris* is the larval stage of *Taenia taeniaeformis* found in the rodent. |
| 25 | El Kady *et al.* [25] | Egypt | February 2007 to January 2008 | *Mus musculus* (F=6, M=9, N=15)  *Rattus norvegicus* (F=15, M=18, N=33)  *Rattus rattus alexandrines* (F=27, M=21, N=48)  *Rattus rattus frugivorous* (F=18, M=21, N=39)  n=135, R=55, Rc=23, Rn=34, Rt=9 | **Cestode**  *Hymenolepis nana*  *Hymenolepis diminuta*  *Cysticercus fasciolaris*  *Mathevotaenia symmetrica*  **Nematode**  *Angiostrongylus cantonensis*  *Capillaria hepatica*  *Protospirura muris*  *Protospirura marsupialis*  *Syphacia muris*  *Syphacia obvelata*  *Strongyloides papillosus*  *Streptopharagus kuntzi*  *Trichuris muris*  *Enterobius minutus*  **Trematode**  *Haplorchis pumilio*  *Haplorchis yokogawai*  *Stictodora tridactyla*  *Echinostoma callawayenis*  *Pygidiopsis genata*  *Echinoparyphium rodentium*  *Mesostephanus rodentium*  *Mesostephanus aegypticus*  *Prosthodendrium ascida*  *Prosthodendrium* sp. |  | *Cysticercus fasciolaris* is the larval stage of *Hydatigera taeniaeformis* (or *Taenia taeniaeformis*) found in the rodent. Spelling corrected from *Pygedispsis genala*  to *Pygidiopsis genata* |
| 26 | el Shazly *et al.* [26] | Egypt, Dakahlia Governorate | - | *Acomys cahirinus cahirinus*  *Arvicanthis niloticus niloticus*  *Gerbillus andersoni*  *Gerbillus pyramidum*  *Mus musculus praetextus*  *Nesokia indica*  *Rattus norvegicus*  *Rattus rattus* | **Cestode**  *Hymenolepis diminuta*  *Hymenolepis nana*  *Cysticercus fasciolaris*  **Nematode**  *Angiostrongylus cantonensis*  *Capillaria hepatica*  *Strongyloides ratti*  *Syphacia muris*  *Syphacia obvelata*  *Trichuris muris*  **Trematode**  *Echinostoma callawayensis*  *Haplorchis pumilio*  *Haplorchis yokogawai*  *Heterophyes heterophyes*  *Stictodora tridactyla* |  | *Cysticercus fasciolaris* is the larval stage of *Taenia taeniaeformis* found in the rodent. |
| 27 | Elshazly *et al.* [27] | Egypt, Dakahlia and Menoufia governorates | February 2007 to January 2008 | *Mus musculus*  *Rattus norvegicus*  *Rattus rattus alexandrinus*  *Rattus rattus frugivorous* | **Cestode**  *Hymenolepis diminuta*  *Hymenolepis nana*  *Mathevotaenia symmetrica*  *Cysticercus fasciolaris*  **Nematode**  *Aspiculuris tetraptera*  *Capillaria hepatica*  *Dermatoxys* spp.  *Enterobius minutus*  *Protospirura muris*  *Protospirura marsupialis*  *Strongyloides ratti*  *Syphacia muris*  *Syphacia obvelata*  *Trichuris muris*  **Trematode**  *Echinoparyphium recurvatum*  *Echinostoma callawayensis*  *Haplorchis pumilio*  *Haplorchis yokogawai*  *Mesostephanus aegypticus*  *Mesostephanus* spp.  *Prosthodendrium ascidia*  *Prosthodendrium* spp.  *Pygidiopsis genata*  *Stictodora tridactyla* | Rodent infestation varies with geographical area. However, geographical area does not have any significance in rodent helminths infestation. | Spelling corrected from *Protosprura marsupialis* to *Protospirura marsupialis*.  *Cysticercus fasciolaris* is the larval stage of *Taenia taeniaeformis* found in the rodent. |
| 28 | El-Shazly *et al.* [28] | Egypt, Dakahlia governorate |  | *Rattus norvegicus* (N=142)  *Rattus rattus* (N=176)  n=318, R=16, Rt=16 | **Trematode**  *Schistosoma* spp. |  |  |
| 29 | Fair *et al.*  [29] | Israel | - | *Microtus guentheri* | **Cestode**  *Andrya rauschi* |  |  |
|  |  | Syria | - | *Spalax ehrenbergi* | **Cestode**  *Paranoplocephala nevoi* |  |  |
| 30 | Fasihi Harandi *et al.* [30] | Iran, Kerman province, Southeast Iran | November 2007-February 2009 | *Dryomys nitedula* (N=1)  *Meriones libycus* (N=7)  *Meriones persicus* (N=21)  *Mus musculus* (N=2)  *Nesokia indica* (N=12)  *Tatera indica* (N=5)  n=48, Rc=5, Rn=15, Ra=1 | **Cestode**  *Hymenolepis diminuta*  *Hymenolepis nana*  **Nematode**  *Trichuris muris*  *Mastophorus muris* |  |  |
| 31 | Garedaghi and Afshin Khaki [31] | Iran, Tabriz, East Azerbaijan Province, Northwest Iran | 2011-2012 | *Mus musculus* (N=8)  *Rattus norvegicus* (N=36)  *Rattus rattus* (N=11)  Unknown type (N=2)  n=57 | **Cestode**  *Hymenolepis diminuta*  *Hymenolepis nana*  **Nematode**  *Trichosomoides crassicauda*  *Gongylonema pulchrum*  *Trichocephala* spp. | Helminths infestation in rodents varies with geographical location as well as rodent species. | Spelling corrected from *Trichocephal* to *Trichocephala* |
| 32 | Gholipoury *et al.* [32] | Iran, Golestan province, Northeast Iran | 2012-2014 | *Meriones libycus* (N=4)  *Mus musculus* (N=48)  *Rattus norvegicus* (N=35)  *Rhombomys opimus* (N=4)  n=91, Rc=7, Rn=16 | **Cestode**  *Cysticercus fasciolaris*  *Hymenolepis diminuta*  **Nematode**  *Angiostrongylus* spp.  *Capillaria* spp.  *Trichuris* spp. | Rattus norvegicus was more prevalent than other rats for the helminths. The helminths have specificity on the location of rodent sampling. |  |
| 33 | Greenberg  [33] | Israel, Jerusalem, Negev, and Galilee, Carmel region | June 1965 - May 1966 | *Acomys cahirinus* (N=178)  *Acomys russatus* (N=20)  *n=198,* | ***Cestodes***  *Hymenolepis diminuta*  *Rodentolepis negevi*  *Pseudandrya monardi*  *Mathevotaenia rodentinum*  *Mesocestoides spp.*  **Nematode**  *Aspiculuris Africana*  *Dentostomella kuntzi*  *Gongylonema* sp.  *Metathelazia acomysi*  *Streptopharagus kutassi*  *Syphacia minuta*  *Trichosomoides crassicauda*  *Trichuris muris*  ***Trematode***  *Scaphiostomum* sp. |  | Tetrathyridium is the larval state of Mesocestoides tape worm |
| 34 | Gurler *et al.* [34] | Turkey, Samsun | - | *Rattus norvegicus*  n=34, R=27, Rc=12, Rn=27 | **Cestode**  *Hymenolepis diminuta*  **Nematode**  *Heterakis spumosa*  *Nippostrongylus brasiliensis*  *Strongyloides ratti* | Helminths prevalence does not depend significantly on rodent sex; however, the helminths were detected only in adults. |  |
| 35 | Haridy *et al.* [35] | Egypt, Giza governorate | - | *Rattus rattus* | **Trematode**  *Fasciola* spp. |  |  |
| 36 | Hasson  [36] | Iraq, Baghdad | 1990-1994 | *Mus musculus* (N=35)  *Rattus norvegicus* (N=89)  *Rattus rattus* (N=62)  n=186 | **Cestode**s  *Cysticercus fasciolaris*  *Hymenolepis diminuta*  *Hymenolepis nana*  *Hymenolepis* sp.  *Mathevotaenia rodentinum*  **Nematode**  *Aspiculuris tetraptera*  *Syphacia obvelata*  *Trichuris* sp. | Rattus norvegicus were found most prevalent for helminths infection. | The common name of *Vampirolepis* sp. is *Hymenolepis* sp. Spelling corrected from *rodentium* to *rodentinum* and *Aspicularis* to *Aspiculuris* |
| 37 | Jones and El-Azazy [37] | Egypt, Sharkia province |  | *Rattus norvegicus* (N=72)  n=72, R=1, Rt=1 | **Trematode**  *Coelomotrema aegyptiaca* |  |  |
| 38 | Kamranrashani *et al.* [38] | Iran | 2010-2011 | *Rhombomys opimus* (F=12, M=65), n=77, R=63, Rc=17, Rn=59 | **Cestode**  *Hymenolepis nana fraterna*  *Skrjabinotaenia lobata*  *Taenia endothoracica*  **Nematode**  *Acanthocheilonema viteae*  *Syphacia muris*  *Trichuris muris*  *Trichuris rhombomidis*  *Trichuris* spp. | Helminths infestation does not differ with rodent sex. | Spelling corrected from *endothoracicus* to *endothoracica*. *Acanthocheilonema viteae* is the modern name of *Dipetalonema viteae* |
| 39 | Khajeh *et al.* [39] | Iran |  | *Acomys dimidiatus* (N=21)  *Apodemus witherbyi* (N=6)  *Calomyscus hotsoni* (N=6)  *Cricetulus migratorius* (N=1)  *Gerbillus nanus* (N=13)  *Golunda ellioti* (N=2)  *Jaculus blandordi* (N=7)  *Meriones libycus* (N=13)  *Meriones mystacinus* (N=1)  *Mus musculus* (N=36)  *Nesokia indica* (N=8)  *Rattus rattus* (N=2)  *Tatera indica* (N=30)  n=146, R=49 | **Cestode**  *Choanotaenia* sp.  *Hymenolepis diminuta*  *Raillieitina* sp.  **Nematode**  *Aspiculuris tetraptera*  *Heligmosomoides skrjabini*  *Labiostomum naimi*  *Labiostomum* sp.  *Mastophorus muris*  *Physaloptera* sp.  *Syphacia obvelata*  *Trichuris muris* |  | Spelling corrected from *Heligmosomodies* to *Heligmosomoides*; *Mastrophorus* to *Mastophorus; tetrptera to tetraptera* |
| 40 | Khalil *et al.* [40] | Kuwait | August 1976 | *Gerbilus cheesmani*  *Meriones crassus* | **Cestode**  *Hymenolepis diminuta*  *Taenia endothoracicus*  **Nematode**  *Abbreviata kuwaitensis* |  |  |
| 41 | Kia *et al.* [41] | Iran, Khuzestan province, Southwest Iran | 1999-2000 | *Mus musculus* (F=12, M=2, N=14)  *Rattus norvegicus* (F=43, M=29, N=72)  *Rattus rattus* (F=3, M=1, N=4)  n=90 | **Cestode**  *Hymenolepis nana*  *Cysticercus fasciolaris*  **Nematode**  *Gongylonema monigi*  *Gongylonema neoplasticum Syphacia muris*  *Strongyloides* sp.  *Trichosomoides crassicauda* | Rattus norvegicus is the most prevalent rat with helminths. None of the helminths except *H. nana* prevalence depends on rodent sex, more in males than a female rat. |  |
| 42 | Kia *et al.* [42] | Iran, Ardabil province, Northwest Iran | 2005-2007 | *Meriones persicus* (F=89, M=71, N=160)  *Microtus socialis* (F=4, M=13, N=17)  n=177, R=131 | **Cestode**  *Hymenolepis diminuta*  *Hymenolepis nana*  *Taenia endothoracicus*  *Cysticercus fasciolaris*  **Nematode**  *Aspiculuris tetraptera*  *Capillaria hepatica*  *Dentostomella translucida*  *Heligmosomum mixtum*  *Mesocestoides* spp.  *Physaloptera* sp.  *Syphacia obvelata*  *Syphacia syphacia*  *Trichuris* spp. | There is no statistical difference between males and females for infectivity with parasites in the rats. | *Cysticercus fasciolaris* is the larval stage of *Taenia taeniaeformis* found in the rodent. |
| 43 | Meshkekar *et al.* [43] | Iran, Teran | 2009-2010 | *Rattus norvegicus* (N=16)  *Rattus rattus* (N=104)  n=120, R=75 | **Cestode**  *Cysticercus fasciolaris*  *Hymenolepis diminuta*  *Hymenolepis nana*  **Nematode**  *Capillaria annulosa*  *Heterakis spumosa* | Helminth occurrence differs with the locality of rodent collection. *R. rattus* was a dominant rodent for helminth. There is no significant association between the prevalence of helminths infection and host sex and maturity. |  |
| 44 | Metwally *et al.* [44] | Saudi Arabia; Makkah and Riyadh | March-April, 2017 | *Rattus norvegicus* (N=120) | **Cestode**  *Hymenolepis nana* |  |  |
| 45 | Mikhail *et al.* [45] | Egypt, Suez, Menoufia,  Giza, Damietta and Beni-Sewaf  Governorates | 2004 - 2005 | *Acomys cahirinus* (n=41)  *Mus musculus* (n=199)  *Rattus norvegicus* (n=464)  *Rattus rattus alexandrines* (n=285)  *Rattus rattus frugivorous* (n=212)  n=1201, Rc=330, Rt=286 | **Cestode**  *Hymenolepis diminuta*  **Nematode**  *Streptopharagus kuntzi* | There was seasonal variation of nematode infestation among rodents. The nematode infestation was more in summer. Cestode and nematode infestation was associated with rodent species. R. norvegicus was more infested with *H. diminuta* and *S. kuntzi* |  |
| 46 | Mirjalali *et al.* [46] | Iran, Razavi Khorasan province, Northeast Iran | 2011 | *Rhombomys opimus* | **Cestode**  *Hymenolepis nana* |  | *Hymenolepis nana* is the modern name of *Rodentolepis nana* |
| 47 | Moradpour *et al.* [47] | Iran, West Iran | March 2015-February 2016 | *Allactaga elater* (N=6)  *Apodemus witherbyi* (N=51)  *Arvicola amphibious* (N=1)  *Calomyscus elburzensis* (N= 21)  *Meriones libycus* (N=13)  *Microtus* spp. (N=70)  *Mus musculus* (N=76)  *Tatera indica* (N=15)  n=253, R=109, Rt=1, Rc=22, Rn=109, Ra=1 | **Cestode**  *Hymenolepis diminuta*  *Hymenolepis nana*  *Mesocestoides* sp.  *Cysticercus fasciolaris*  **Nematode**  *Aspiculuris tetraptera*  *Capillaria* sp.  *Gongylonema* sp.  *Heligmosomoides polygyrus*  *Nippostrongylus brasiliensis*  *Physaloptera* sp.  *Syphacia obvelata*  *Trichuris muris*  **Trematode**  *Notocotylus neyrai* | *S. obvelata* was the most abundant helminth, particularly in *Mus musculus*. | Spelling corrected from *Aspicularis* to *Aspiculuris*.  *Cysticercus fasciolaris* is the larval stage of *Taenia taeniaeformis* found in the rodent. |
| 48 | Morsy *et al.* [48] | Egypt, Ismailiya Governorate | 1981 | *Mus musculus* (N=50)  *Rattus norvegicus* (N=980)  *Rattus rattus* (N=344)  n=1374 | **Cestode**  *Echinococcus* sp.  **Nematode**  *Trichinella* sp. |  |  |
| 49 | Mowlavi *et al.* [49] | Iran, Ismailiya Governorate | 2000-2010 | *Rattus norvegicus*  n=500, R=1, Rt=1 | **Trematode**  *Plagiorchis muris* |  |  |
| 50 | Nateghpour *et al.* [50] | Iran, Tehran and Bandar Anzali | - | *Gerbillus nanus* (N=5)  *Meriones hurrianae* (N=44)  *Meriones libycus* (N=4)  *Tatera indica* (N=47)  n=100, R=40, Rc=19, Rn=21 | **Cestode**  *Hymenolepis diminuta*  *Hymenolepis nana fraterna*  *Skrjabinotaenia* sp.  **Nematode**  *Rictularia* spp.  *Trichostrongylus* spp.  *Trichuris trichiura* |  | Spelling corrected from *hurriana* to *hurrianae* |
| 51 | Pakdel *et al.* [51] | Iran, Sistan and Baluchistan Province, Southeast Iran | January-October, 2011 | *Mus musculus* (F=43, M=67, N=110)  *Rattus norvegicus* (F=10, M=13, N=23)  *Rattus rattus* (F=1, M=4, N=5)  n=138, R=58 | **Cestode**  *Cysticercus fasciolaris*  *Hymenolepis diminuta*  **Nematode**  *Aspiculuris tetraptera*  *Capillaria hepatica*  *Heterakis spumosa*  *Syphacia muris*  *Syphacia obvelata*  *Trichuris muris* | Rodents are more infected with nematodes than helminths. |  |
| 52 | Ranjbar *et al.* [52] | Iran, Kohgiluyeh and Boyer-Ahmad Province, Southwest Iran | January-November, 2014 | *Apodemus sylvaticus* (N=10)  *Arvicola terrestris* (N=1)  *Calomyscus bailwardi* (N=1)  *Meriones persicus* (N=25)  *Rattus norvegicus* (N=8)  *Rattus rattus* (N=7)  n=52, R=38 | **Cestode**  *Anoplocephalidae* sp.  *Cysticercus fasciolaris*  *Hymenolepis diminuta*  *Hymenolepis nana fraterna*  *Skrjabinotaenia* sp.  **Nematode**  *Aspiculuris tetraptera*  *Gongylonema* sp.  *Rictularia* sp.  *Syphacia* sp.  *Trichostrongylus* sp.  *Trichuris muris* | Helminth infectivity has no association with rodent sex and weight of rodents. | Spelling corrected from *terresterris* to *terrestris*. |
| 53 | Sadighian *et al.* [53] | Iran |  | *Rhombomys opimus* | **Nematode**  *Trichuris mofidii* |  |  |
| 54 | Saoud *et al.* [54] | Egypt, Eastern Delta | 1981-1982 | *Acomys cahirinus* (F=14, M=24, N=38)  *Arvicanthis niloticus niloticus* (F=191, M=307, N=498)  *Rattus norvegicus* (F=117, M=169, N=286)  *Rattus rattus* (F=24, M=30, N=54)  n=876, R=309, Rt=15, Rc=253, Rn=68, Ra=4 | **Cestode**  *Andrya* sp.  *Taenia taeniformis*  *Hymenolepis* diminuta  **Nematode**  *Arpiculuris* sp.  *Syphacia* obvelata  **Trematode**  *Echinochasmus* sp.  *Echoinostoma* sp.  *Plagiorchis* sp. |  |  |
| 55 | Soliman *et al.* [55] | Egypt, St Katherine, South Sinai | April-May, 2003 | *Acomys dimidiatus*  n=47, R=26, Rn=26 | **Nematode**  *Aspiculuris* spp.  *Dentostomella* spp.  **Nematode**  *Syphacia* spp. | There is spatial variation in the distribution of the nematode community. | Spelling corrected from *Aspicularis* to *Aspiculuris* |
| 56 | Sures *et al.*  [56] | Egypt, Cairo | March-April, 2000 | Rat  n=40, R=32 | **Cestode**  *Hymenolepis diminuta*  *Cysticercus fasciolaris* |  | *Cysticercus fasciolaris* is the larval stage of *Taenia taeniaeformis* found in the rodent. |
| 57 | Sursal *et al.* [57] | Turkey, Central Anatolia | September 2009-March, 2010 | Dwarf hamster (N=33)  Syrian hamster (N=71)  n=104, Rc=8, Rn=35 | **Cestode**  *Hymenolepis nana*  **Nematode**  *Aspiculuris* spp.  *Syphacia* spp.  *Trichuris* spp. |  | *Mesocricetus auratus* is Syrian hamster*. Hymenolepis nana* is modern name of *Rodentolepis nana* |
| 58 | Wanas *et al.* [58] | Egypt |  | *Acomys cahirinus*  *Arvicanthis niloticus*  *Gerbillus gerbillus*  *Mus musculus*  *Rattus norvegicus*  *Rattus rattus* | **Cestode**  *Cysticercus fasciolaris* |  | Hydatigera taeniaeformis is the synonym of *T. taeniaeformis.* The cystic form is *Cysticercus fasciolaris* that is found in rodent. |
| 59 | Wanas *et al.* [59] | Egypt. El-Giza governorate |  | *Arvicanthis niloticus*  *Gerbillus gerbillus*  *Rattus norvegicus*  *Rattus rattus* | **Nematode**  *Streptopharagus kuntzi*  *Protospirura marsupialis*  *Protospirura muris* |  |  |
| 60 | Wertheim and Chabaud [60] | Israel, Ein Yahav, Negev |  | *Acomys cahirinus*  *Gerbillus dasyurus*  *Gerbillus gerbillus*  *Gerbillus pyramidum*  *Meriones crassus* | **Nematode**  *Skrjabinocapillaria rodentium* |  |  |
| 61 | Wertheim and Giladi [61] | Israel, Ein Yahav, Negev |  | *Gerbillus dasyurus*  *Meriones crassus* | **Nematode**  *Pneumospirura rodentium* |  |  |
| 62 | Wertheim  [62] | Israel, Ein Yahav, Negev |  | *Spalax ehrenbergi* | **Cestode**  *Paranoplocephala* sp.  **Nematode**  *Heligmonella* sp.  *Trichuris muris*  *Ganguleterakis spalaxi*  *Gongylonema longispiculum* |  |  |
| 63 | Yousefi *et al.* [63] | Iran, Hamadan, Northeast Iran | February 2010-November, 2012 | *Apodemus sylvaticus* (F=23, M=37, N=60)  *Mus musculus* (F=38, M=34, N=72)  n=132, R=54 | **Cestode**  *Anoplocephalidae* sp.  *Cysticercus fasciolaris*  *Hymenolepis diminuta*  *Hymenolepis nana*  *Rodentolepis crassa*  *Skrjabinotaenia lobata*  **Nematode**  *Syphacia fredrici*  *Syphacia obvelata*  *Syphacia ohtarom*  *Syphacia stroma*  **Trematode**  *Plagiorchis muris*  *Protospirura muricola* | There was no rodent sex effect on helminth infestation |  |
| 64 | Yousif and Ibrahim [64] | Egypt, Giza, Qalyoubia, Menoufia governorate |  | *Arvicanthis niloticus* (N=38)  *Rattus norvegicus* (N=55)  *Rattus rattus* (N=65)  n=158 | **Nematode**  Angiostrongylus cantonensis | Only *R. norvegicus* was found positive with *A. cantonensis* |  |
| 65 | Zarei *et al.* [65] | Iran, Ardabil province, Northwest Iran | April 2014- March 2015 | *Cricetulus migratorius* (F=8, M=16, N=24)  *Meriones persicus* (F=49, M=69, N=118)  *Mus musculus* (F=22, M=41, N=63)  n=205, R=152 | **Cestode**  *Cysticercus fasciolaris*  *Hymenolepis diminuta*  *Hymenolepis nana fraterna*  *Paranoplocephala* sp.  *Mesocestoides spp.*  *Taenia endothoracica*  **Nematode**  *Aspiculuris tetraptera*  *Capillaria hepatica*  *Heligmosomum* sp.  *Streptopharagus kuntzi*  *Syphacia fredrici*  *Trichuris rhombomidis* |  | Spelling corrected from *endothoracicus* to endothoracica*;* Tetrathyridium is the larval stage of *Mesocestoides spp.* |

N: Total number in a species, f: Total females, m: Total males, n: total number in a study, R: Total rodents infested with endoparasites, Rc: Total rodents infested with cestodes, Rn: Total rodents infested with nematodes, Rt: Total rodents infested trematodes

**Reference**

1. Abd el-Wahed, M.M.; Salem, G.H.; El-Assaly, T.M. The role of wild rats as a reservoir of some internal parasites in Qalyobia governorate. *J. Egypt. Soc. Parasitol.* **1999**, *29*, 495–503.
2. Abdel-Gaber, R.; Abdel-Ghaffar, F.; Al Quraishy, S.; Morsy, K.; Saleh, R.; Mehlhorn, H. Morphological Re-Description and 18 S rDNA Sequence Confirmation of the Pinworm Aspiculuris tetraptera (Nematoda, Heteroxynematidae) Infecting the Laboratory Mice *Mus musculus*. *J. Nematol.* **2018**, *50*, 117–132, doi:10.21307/jofnem-2018-026.
3. Abdel-Gaber, R. Syphacia obvelata (Nematode, Oxyuridae) infecting laboratory mice *Mus musculus* (Rodentia, *Muridae*): Phylogeny and host-parasite relationship. *Parasitol. Res.* **2016**, *115*, 975–985, doi:10.1007/s00436-015-4825-0.
4. Abdel-Salam, F.A.; Galal, A.A.; Ali, M.K. The Role of Rodents as a Reservoir of Zoonotic Intestinal Parasites at Sohag Governorate, Egypt. *Assiut Vet. Med. J.* **1994**, *30*, 14.
5. Abou-Zinadah, N.Y.; Fouad, M.A. Anti-Fasciola antibodies among rodents and sheep in Jeddah, Saudi Arabia. *J. Egypt. Soc. Parasitol.* **2005**, *35*, 711–716.
6. Abu-Madi, M.A.; Behnke, J.M.; Mikhail, M.; Lewis, J.W.; Al-Kaabi, M.L. Parasite populations in the brown rat *Rattus norvegicus* from Doha, Qatar between years: The effect of host age, sex and density. *J. Helminthol.* **2005**, *79*, 105–111, doi:10.1079/JOH2005274.
7. Abu-Madi, M.A.; Lewis, J.W.; Mikhail, M.; El-Nagger, M.E.; Behnke, J.M. Monospecific helminth and arthropod infections in an urban population of brown rats from Doha, Qatar. *J. Helminthol.* **2001**, *75*, 313–320.
8. Al Hindi, A.I.; Abu-Haddaf, E. Gastrointestinal parasites and ectoparasites biodiversity of *Rattus rattus* trapped from Khan Younis and Jabalia in Gaza strip, Palestine. *J. Egypt. Soc. Parasitol.* **2013**, *43*, 259–268, doi:10.12816/0006382.
9. Allymehr, M.; Tavassoli, M.; Manoochehri, M.H.; Ardavan, D. Ectoparasites and Gastrointestinal Helminths of House Mice (*Mus musculus*) from Poultry Houses in Northwest Iran. *Comp. Parasitol.* **2012**, *79*, 283–287, doi:10.1654/4534.1.
10. Al-Quraishy, S.; Gewik, M.M.; Abdel-Baki, A.A. The intestinal cestode *Hymenolepis diminuta* as a lead sink for its rat host in the industrial areas of Riyadh, Saudi Arabia. *Saudi J. Biol. Sci.* **2014**, *21*, 387–390, doi:10.1016/j.sjbs.2013.09.010.
11. Al-Qureishy, S. Lead concentrations in some organs of the rat *Meriones libycus* and its parasite *Hymenolepis diminuta* from Riyadh City, KSA. *J. Egypt. Soc. Parasitol.* **2008**, *38*, 351–358.
12. Antoniou, M.; Psaroulaki, A.; Toumazos, P.; Mazeris, A.; Ioannou, I.; Papaprodromou, M.; Georgiou, K.; Hristofi, N.; Patsias, A.; Loucaides, F.; et al. Rats as Indicators of the Presence and Dispersal of Pathogens in Cyprus: Ectoparasites, Parasitic Helminths, Enteric Bacteria, and Encephalomyocarditis Virus. *Vector-Borne Zoonotic Dis.* **2010**, *10*, 867–873, doi:10.1089/vbz.2009.0123.
13. Arzamani, K.; Salehi, M.; Mobedi, I.; Adinezade, A.; Hasanpour, H.; Alavinia, M.; Darvish, J.; Shirzadi, M.R.; Mohammadi, Z. Intestinal Helminths in Different Species of Rodents in North Khorasan Province, Northeast of Iran. *Iran. J. Parasitol.* **2017**, *12*, 267–273.
14. Ashour, A.A.; Lewis, J.W. *Gongylonema aegypti* n. sp. (Nematoda: Thelaziidae) from Egyptian rodents. *Syst. Parasitol.* **1986**, *8*, 199–206, doi:10.1007/BF00009888.
15. Avcioglu, H.; Guven, E.; Balkaya, I.; Kirman, R.; Bia, M.M.; Gulbeyen, H.; Kurt, A.; Yaya, S.; Demirtas, S. First detection of *Echinococcus multilocularis* in rodent intermediate hosts in Turkey. *Parasitology* **2017**, *144*, 1821–1827, doi:10.1017/s0031182017001226.
16. Azzam, K.M.; Abd El-Hady, E.A.; El-Abd, N. Survey of Natural Infection with *Echinostoma liei* in Aquatic Snails and Wild Rodents in Egypt. *Egypt. J. Biol. Pest Control* **2015**, *25*, 427–432.
17. Azzam, K.M.; El-Abd, N.M.; Abd El-Hady, E.A. Survey of endoparasites of different rodent species in Egypt. *Egypt. J. Biol. Pest Control* **2016**, *26*, 815–820.
18. Behnke, J.M.; Barnard, C.J.; Mason, N.; Harris, P.D.; Sherif, N.E.; Zalat, S.; Gilbert, F.S. Intestinal helminths of spiny mice (*Acomys cahirinus dimidiatus*) from St Katherine’s Protectorate in the Sinai, Egypt. *J. Helminthol.* **2000**, *74*, 31–43.
19. Behnke, J.M.; Harris, P.D.; Bajer, A.; Barnard, C.J.; Sherif, N.; Cliffe, L.; Hurst, J.; Lamb, M.; Rhodes, A.; James, M.; et al. Variation in the helminth community structure in spiny mice (*Acomys dimidiatus*) from four montane wadis in the St Katherine region of the Sinai Peninsula in Egypt. *Parasitology* **2004**, *129*, 379–398, doi:10.1017/S003118200400558X.
20. Beiromvand, M.; Akhlaghi, L.; Fattahi Massom, S.H.; Meamar, A.R.; Darvish, J.; Razmjou, E. Molecular Identification of *Echinococcus multilocularis* Infection in Small Mammals from Northeast, Iran. *PLoS Negl. Trop. Dis.* **2013**, *7*, e2313, doi:10.1371/journal.pntd.0002313.
21. Borji, H.; Khoshnegah, J.; Razmi, G.; Amini, H.; Shariatzadeh, M. A survey on intestinal parasites of golden hamster (*Mesocricetus auratus*) in the northeast of Iran. *J. Parasit. Dis.* **2014**, *38*, 265–268, doi:10.1007/s12639-013-0238-0.
22. Celebi, B.; Taylan Ozkan, A.; Babur, C. Capillaria Hhepatica in mouse (*Apodemus flavicollis*) from Giresun Province of Turkey. *Turk. Parazitolojii Derg.* **2014**, *38*, 208–210, doi:10.5152/tpd.2014.3501.
23. Ebrahimi, M.; Sharifi, Y.; Nematollahi, A. Assessment of gastrointestinal helminths among house mice (*Mus musculus*) caught in the north-west of Iran, with a special view on zoonotic aspects. *Comp. Clin. Pathol.* **2016**, *25*, 1047–1051, doi:10.1007/s00580-016-2308-z.
24. El Gindy, M.S.; Morsy, T.A.; Bebars, M.A.; Sarwat, M.A.; Arafa, M.A.; Salama, M.M. Rodents as reservoir of zoonotic intestinal helminths in Suez Canal Zone with the possible immunological changes. *J. Egypt. Soc. Parasitol.* **1987**, *17*, 259–273.
25. El Kady, G.A.; Gheneam, Y.M.; Bahgat, I.M. Zoonotic helminthes of commensal rodents in Talkha Center, Dakahlia Governorate. *J. Egypt. Soc. Parasitol.* **2008**, *38*, 863–872.
26. El Shazly, A.M.; Morsy, T.A.; el Kady, G.A.; Ragheb, D.A.; Handousa, A.E.; Ahmed, M.M.; Younis, T.A.; Habib, K.S. The helminthic parasites of rodents in Dakahlia Governorate, with reference to their Egyptian helminth fauna. *J. Egypt. Soc. Parasitol.* **1994**, *24*, 413–428.
27. Elshazly, A.M.; Awad, S.I.; Azab, M.S.; Elsheikha, H.M.; Abdel-Gawad, A.G.; Khalil, H.H.; Morsy, T.A. Helminthes of synanthropic rodents (Rodentia: *Muridae*) from Dakahlia and Menoufia, Egypt. *J. Egypt. Soc. Parasitol.* **2008**, *38*, 727–740.
28. el-Shazly, A.M.; Romia, S.A.; el Ganayni, G.A.; Abou-Zakham, A.A.; Sabry, A.H.; Morsy, T.A. Antibodies against some zoonotic parasites in commensal rodents trapped from Dakahlia Governorate, Egypt. *J. Egypt. Soc. Parasitol.* **1991**, *21*, 169–177.
29. Fair, J.M.; Schmidt, G.D.; Wertheim, G. New species of Andrya and Paranoplocephala (Cestoidea: *Anoplocephalidae*) from voles and mole-rats in Israel and Syria. *J. Parasitol.* **1990**, *76*, 641–644, doi:10.2307/3282975.
30. Fasihi Harandi, M.; Madjdzadeh, S.M.; Ahmadinejad, M. Helminth parasites of small mammals in Kerman province, southeastern Iran. *J. Parasit. Dis.* **2016**, *40*, 106–109, doi:10.1007/s12639-014-0456-0.
31. Garedaghi, Y.; Afshin Khaki, A. Prevalence of Gastrointestinal and Blood Parasites of Rodents in Tabriz, Iran, with Emphasis on Parasitic Zoonoses. *Crescent J. Med Biol. Sci.* **2014**, *1*, 9–12.
32. Gholipoury, M.; Rezai, H.R.; Namroodi, S.; Arab Khazaeli, F. Zoonotic and Non-zoonotic Parasites of Wild Rodents in Turkman Sahra, Northeastern Iran. *Iran. J. Parasitol.* **2016**, *11*, 350–357.
33. Greenberg, Z. Helminths of mammals and birds of israel i. *Helminths of acomys* spp. (rodentia, murinae). *Isr. J. Zool.* **1969**, *18*, 25–38, doi:10.1080/00212210.1969.10688272.
34. Gurler, A.T.; Beyhan, Y.E.; Bolukbas, C.S.; Acici, M.; Umur, S. Gastro-intestinal helminths of wild rats (brown rat-*Rattus norvegicus*, Berkenhout 1769) in Samsun, Turkey. *Ank. Univ. Vet. Fak. Derg.* **2011**, *58*, 289–290, doi:10.1501/Vetfak_0000002490.
35. Haridy, F.M.; Morsy, T.A.; Ibrahim, B.B.; Abdel Gawad, A.G.; Mazyad, S.A. *Rattus rattus*: A new host for fascioliasis. *J. Egypt. Soc. Parasitol.* **2003**, *33*, 647–648.
36. Hasson, R.H. Zoonotic &Nonzoonotic Endoparasites of Rodents from Some Districts in Baghdad. *Diyala J. Pure Sci.* **2010**, *6*, 11.
37. Jones, A.; El-Azazy, O.M.E. *Coelomotrema aegyptiaca* sp, nov., an unusual prosthogonimid trematode from *Rattus norvegicus* (berkenhout) in Egypt. *J. Nat. Hist.* **1986**, *20*, 707–712, doi:10.1080/00222938600770481.
38. Kamranrashani, B.; Kia, E.; Mobedi, I.; Mohebali, M.; Zarei, Z.; Mowlavi, G.; Hajjaran, H.; Abai, M.; Sharifdini, M.; Kakooei, Z.; et al. Helminth Parasites of *Rhombomys opimus* from Golestan Province, Northeast Iran. *Iran. J. Parasitol.* **2013**, *8*, 78–84.
39. Khajeh, A.; Mohammadi, Z.; Darvish, J.; Razmi, G.R.; Ghorbani, F.; Mohammadi, A.; Mobedi, I.; Shahrokhi, A.R. A survey on endoparasites in wild rodents of the Jaz Murian depression and adjacent areas, southeast of Iran. *J. Parasit. Dis.: Off. Organ Indian Soc. Parasitol.* **2018**, *42*, 589–597, doi:10.1007/s12639-018-1040-9.
40. Khalil, L.F.; Hassounah, O.; Behbehani, K. Helminth parasites of rodents in Kuwait with the description of a new species *Abbreviata kuwaitensis* (Nematoda: *Physalopteridae*). *Syst. Parasitol.* **1979**, *1*, 67–73, doi:10.1007/BF00009775.
41. Kia, E.B.; Homayouni, M.M.; Farahnak, A.; Mohebai, M.; Shojai, S. Study of Endoparasites of Rodents and their Zoonotic Importance in Ahvaz, South West Iran. *Iran. J. Public Health* **2001**, *30*, 4.
42. Kia, E.; Shahryary-Rad, E.; Mohebali, M.; Mahmoudi, M.; Mobedi, I.; Zahabiun, F.; Zarei, Z.; Miahipoor, A.; Mowlavi, G.; Akhavan, A.; et al. Endoparasites of rodents and their zoonotic importance in germi, dashte-mogan, ardabil province, Iran. *Iran. J. Parasitol.* **2010**, *5*, 15–20.
43. Meshkekar, M.; Sadraei, J.; Mahmoodzadeh, A.; Mobedi, I. Helminth Infections in *Rattus ratus* and *Rattus norvigicus* in Tehran, Iran. *Iran. J. Parasitol.* **2014**, *9*, 548–552.
44. Metwally, D.M.; Al-Enezy, H.A.; Al-Turaiki, I.M.; El-Khadragy, M.F.; Yehia, H.M.; Al-Otaibi, T.T. Gene-based molecular characterization of cox1 and pnad5 in *Hymenolepis nana* isolated from naturally infected mice and rats in Saudi Arabia. *Biosci. Rep.* **2019**, *39*, doi:10.1042/bsr20181224.
45. Mikhail, M.W.; Metwally, A.M.; Allam, K.A.; Mohamed, A.S. Rodents as reservoir host of intestinal helminthes in different Egyptian agroecosystems. *J. Egypt. Soc. Parasitol.* **2009**, *39*, 633–640.
46. Mirjalali, H.; Kia, E.B.; Kamranrashani, B.; Hajjaran, H.; Sharifdini, M. Molecular analysis of isolates of the cestode *Rodentolepis nana* from the great gerbil, *Rhombomys opimus*. *J. Helminthol.* **2016**, *90*, 252–255, doi:10.1017/s0022149x15000115.
47. Moradpour, N.; Borji, H.; Darvish, J.; Moshaverinia, A.; Mahmoudi, A. Rodents Helminth Parasites in Different Region of Iran. *Iran. J. Parasitol.* **2018**, *13*, 275–284.
48. Morsy, T.A.; Michael, S.A.; Bassili, W.R.; Saleh, M.S. Studies on rodents and their zoonotic parasites, particularly leishmania, in Ismailiya Governorate, A.R. Egypt. *J. Egypt. Soc. Parasitol.* **1982**, *12*, 565–585.
49. Mowlavi, G.; Mobedi, I.; Abedkhojasteh, H.; Sadjjadi, S.M.; Shahbazi, F.; Massoud, J. Plagiorchis muris (Tanabe, 1922) in *Rattus norvegicus* in Iran. *Iran. J. Parasitol.* **2013**, *8*, 486–490.
50. Nateghpour, M.; Motevalli-Haghi, A.; Akbarzadeh, K.; Akhavan, A.A.; Mohebali, M.; Mobedi, I.; Farivar, L. Endoparasites of wild rodents in Southeastern Iran. *J. Arthropod-Borne Dis.* **2014**, *9*, 1–6.
51. Pakdel, N.; Naem, S.; Rezaei, F.; Chalehchaleh, A.A. A survey on helminthic infection in mice (*Mus musculus*) and rats (*Rattus norvegicus* and *Rattus rattus*) in Kermanshah, Iran. *Vet. Res. Forum: Int. Q. J.* **2013**, *4*, 105–109.
52. Ranjbar, M.J.; Sarkari, B.; Mowlavi, G.R.; Seifollahi, Z.; Moshfe, A.; Abdolahi Khabisi, S.; Mobedi, I. Helminth Infections of Rodents and Their Zoonotic Importance in Boyer-Ahmad District, Southwestern Iran. *Iran. J. Parasitol.* **2017**, *12*, 572–579.
53. Sadighian, A.; Ghadirian, E.; Sadjadpour, E. Two new species of nematodes of lagomorphs and rodents from Iran. *J. Helminthol.* **1974**, *48*, 241–245, doi:10.1017/S0022149X00022926.
54. Saoud, M.F.; Ramadan, M.M.; Ashour, A.A.; Shahawy, A.A. On the helminth parasites of rodents in the Eastern Delta. I. General survey. *J. Egypt. Soc. Parasitol.* **1986**, *16*, 197–209.
55. Soliman, M.F.; Ibrahim, M.M.; Zalat, S.M. Gastrointestinal nematode community of spiny mice (*Acomys dimidiatus*) from St. Katherine, South Sinai, Egypt. *J. Parasit. Dis.: Off. Organ Indian Soc. Parasitol.* **2015**, *39*, 705–711, doi:10.1007/s12639-013-0410-6.
56. Sures, B.; Scheible, T.; Bashtar, A.R.; Taraschewski, H. Lead concentrations in *Hymenolepis diminuta* adults and *Taenia taeniaeformis* larvae compared to their rat hosts (*Rattus norvegicus*) sampled from the city of Cairo, Egypt. *Parasitology* **2003**, *127*, 483–487, doi:10.1017/S0031182003003901.
57. Sursal, N.; Gokpinar, S.; Yildiz, K. Prevalence of intestinal parasites in hamsters and rabbits in some pet shops of Turkey. *Turk. Parazitolojii Derg.* **2014**, *38*, 102–105, doi:10.5152/tpd.2014.3338.
58. Wanas, M.Q.; Shehata, K.K.; Rashed, A.A. Larval occurrence of Hydatigera taeniaeformis Batsch (1786) (Cestoda: *Taeniidae*) in the liver of wild rodents in Egypt. *J. Egypt. Soc. Parasitol.* **1993**, *23*, 381–388.
59. Mohamed, K.; Wanas, Q.A.; Shehata, K.K.; Rashed, A.A. Studies on the nematode parasites from Egyptian rodents. I. *Spirurid nematodes*. *J. Egypt. Soc. Parasitol.* **1993**, *23*, 851–863.
60. Wertheim, G.; Chabaud, A.G. Helminths of birds and mammals of Israel. VIII.—*Skrjabinocapillaria rodentium* n. sp. (*Nematoda Capillariidae*) from gerbillid and murid rodents. *Ann. Parasitol. Hum. Comparée* **1979**, *54*, 65–68, doi:10.1051/parasite/1979541065.
61. Wertheim, G.; Giladi, M. Helminths of birds and mammals of Israel. VII.*—Pneumospirura rodentium* n. sp. (Pneumospiruridae*—*Thelazioidea). *Ann. Parasitol. Hum. Comparée* **1977**, *52*, 643–646, doi:10.1051/parasite/1977526643.
62. Wertheim, G. Helminths of Birds and Mammals from Israel: III. Helminths from Chromosomal Forms of the Mole-Rat, *Spalax ehrenbergi*. *J. Helminthol.* **1971**, *45*, 161–169, doi:10.1017/S0022149X00007045.
63. Yousefi, A.; Eslami, A.; Mobedi, I.; Rahbari, S.; Ronaghi, H. Helminth Infections of House Mouse (*Mus musulus*) and Wood Mouse (*Apodemus sylvaticus*) from the Suburban Areas of Hamadan City, Western Iran. *Iran. J. Parasitol.* **2014**, *9*, 511–518.
64. Yousif, F.; Ibrahim, A. The first record of *Angiostrongylus cantonensis* from Egypt. *Z. Parasitenkd. Parasitol. Res.* **1978**, *56*, 73–80, doi:10.1007/BF00925940.
65. Zarei, Z.; Mohebali, M.; Heidari, Z.; Davoodi, J.; Shabestari, A.; Haghi, A.M.; Khanaliha, K.; Kia, E.B. Helminth infections of *Meriones persicus* (Persian jird), *Mus musculus* (house mice) and *Cricetulus migratorius* (grey ham-ster): A cross-sectional study in Meshkin-Shahr district, north-west Iran. *Iran. J. Parasitol.* **2016**, *11*, 213–220.
