# Supplementary material for: Helminth Parasites among Rodents in the Middle East Countries: A Systematic Review and Meta-Analysis
Source: Animals (Basel). 2020 Dec 9;10(12):2342. doi: 10.3390/ani10122342 (PMC7764038; doi:10.3390/ani10122342)
Supplement: Supplementary file 1 [file animals-10-02342-s001.zip › Supplimentary file/Supplimentary Table S3.docx]

**Table S3** Cestodes, nematodes, and trematodes in prevailing rodents in the Middle East.

**Table S3a** Prevailing rodents in the Middle East.

| **1. Family:** Calomyscidae  *Calomyscus bailwardi* (Iran), *Calomyscus elburzensis* (Iran), *Calomyscus hotsoni* (Iran), *Calomyscus mystax* (Iran) |
| --- |
| **2. Family:** Cricetidae  *Arvicola amphibius* (Iran), *Arvicola terresteris* (Iran), *Cricetulus migratorius* (Iran), *Ellobius fuscocapillus* (Iran), *Mesocricetus auratus* (Egypt, Iran, Turkey), *Microtus guentheri* (Israel), *Microtus paradoxus* (Iran), *Microtus socialis* (Iran), *Microtus* spp. (Iran, Turkey), *Microtus transcaspicus* (Iran) |
| **3. Family:** Dipodidae  *Allactaga elater* (Iran), *Jaculus blandordi* (Iran), *Jaculus jaculus* (Egypt, Saudi Arabia) |
| **4. Family:** Gliridae  *Dryomys nitedula* (Iran) |
| **5. Family:** Muridae  *Acomys cahirinus* (Egypt, Israel), *Acomys dimidiatus* (Egypt, Iran, Saudi Arabia), *Acomys rassatus* (Israel), *Apodemus flavicollis* (Turkey), *Apodemus* spp. (Turkey), *Apodemus sylvaticus* (Iran), *Apodemus witherbyi* (Iran), *Arvicanthis niloticus* (Egypt), *Gerbillus andersoni* (Egypt), *Gerbillus cheesmani* (Kuwait), *Gerbillus dasyurus* (Israel), *Gerbillus gerbillus* (Egypt, Israel), *Gerbillus nanus* (Iran), *Gerbillus pyramidum* (Egypt, Israel), *Golunda ellioti* (Iran), *Meriones crassus* (Israel, Kuwait, Saudi Arabia), *Meriones hurrianae* (Iran), *Meriones libycus* (Iran, Saudi Arabia), *Meriones mystacinus* (Iran), *Meriones persicus* (Iran), *Mus domesticus* (Egypt), *Mus musculus* (Cyprus, Egypt, Iran, Iraq), *Nesokia indica* (Egypt, Iran), *Rattus norvegicus* (Cyprus, Egypt, Iran, Iraq, Qatar, Saudi Arabia, Turkey), *Rattus rattus* (Cyprus, Egypt, Iran, Iraq, Palestine, Saudi Arabia, Egypt), *Rhombomys opimus* (Iran), *Tatera indica* (Iran) |
| **6. Family:** Spalacidae  *Spalax ehrenbergi* (Israel, Syria) |

**Table S3b** Prevailing cestodes in rodents of the Middle East

| **1. Family:** Anoplocephalidae  *Andrya neotomae* (Egypt), *Andrya rauschi* (Israel), *Andrya* sp. (Egypt), *Anoplocephalidae* sp. (Iran), *Mathevotaenia rodentinum* (Iraq, Israel), *Mathevotaenia symmetrica* (Egypt), *Paranoplocephala nevoiI* (Syria), *Paranoplocephala* sp. (Israel, Iran), *Witenbergitaenia* sp. (Egypt) |
| --- |
| **2. Family:** Catenotaeniidae  *Skrjabinotaenia lobate* (Iran), *Skrjabinotaenia* sp. (Iran) |
| **3. Family:** Davaineidae  *Raillieitina* spp. (Iran, Egypt) |
| **4. Family:** Dilepididae  *Choanotaenia* sp. (Iran) |
| **5. Family:** Dipylidiidae  *Joyeuxiella rossicum* (Egypt) |
| **6. Family:** Hymenolepididae  *Hymenolepis diminuta* (Cyprus, Egypt, Iran, Iraq, Israel, Kuwait, Palestine, Qatar, Saudi Arabia, Turkey), *Hymenolepis nana* (Egypt, Iran, Iraq, Saudi Arabia, Turkey), *Hymenolepis* spp. (Iraq), *Pseudandrya monardi* (Israel), *Rodentolepis crassa* (Iran), *Rodentolepis negevi* (Egypt, Israel) |
| **7. Family:** Mesocestoididae  *Mesocestoides* sp. (Egypt, Iran, Israel) |
| **8. Family:** Taeniidae  *Cysticercus fasciolaris* (Cyprus, Egypt, Iran, Iraq), *Echinococcus multilocularis* (Iran, Turkey), *Echinococcus* sp. (Egypt), *Hydatigera* sp. (Egypt), *Taenia endothoracica* (Iran, Kuwait), *Taenia* spp. (Iran) |

**Table S3c** Prevailing nematodes in rodents of the Middle East.

| **1. Family:** Angiostrongylidae  *Angiostrongylus cantonensis* (Egypt, Israel), *Angiostrongylus* spp. (Iran) |
| --- |
| **2. Family:** Capillaridae  *Skrjabinocapillaria rodentium* (Israel) |
| **3. Family:** Heligmonellidae  *Heligmonella* sp. (Israel), *Heligmonoides josephi* (Palestine), *Nippostrongylus brasiliensis* (Iran, Turkey) |
| **4. Family:** Heligmosomidae  *Heligmosomum mixtum* (Iran), *Heligmosomum* sp. (Iran) |
| **5. Family:** Heterakidae  *Heterakis spumosa* (Iran, Turkey) |
| **6. Family:** Heteroxynematidae  *Arpiculuris* sp. (Egypt, Iran), *Aspiculuris africana* (Egypt, Israel), *Aspiculuris* spp. (Egypt, Turkey), *Aspiculuris tetraptera* (Egypt, Iran, Iraq), *Labiostomum naimi* (Iran), *Labiostomum* sp. (Iran) |
| **7. Family:** Kiwinematidae  *Ganguleterakis spalaxi* (Israel) |
| **8. Family:** Onchocercidae  *Acanthocheilonema viteae* (Iran) |
| **9. Family:** Oxyuridae  *Dermatoxys* spp. (Egypt), *Enterobius minutus* (Egypt), *Enterobious vermicularis* (Egypt), *Syphacia fredrici* (Iran), *Syphacia minuta* (Egypt, Israel), *Syphacia muris* (Egypt, Iran), *Syphacia obvelata* (Egypt, Iran, Iraq, Palestine), *Syphacia ohtarom* (Iran), *Syphacia* spp. (Egypt, Iran, Turkey), *Syphacia stroma* (Iran), *Syphacia syphacia* (Iran) |
| **10. Family:** Oxyuroidea  *Dentostomella kuntzi* (Egypt, Israel), *Dentostomella* spp. (Egypt), *Dentostomella translucida* (Iran) |
| **11. Family:** Physalopteridae  *Abbreviata kuwaitensis* (Kuwait), *Physaloptera* spp. (Cyprus, Iran) |
| **12. Family:** Pneumospiruridae  *Metathelazia acomysi* (Israel), *Pneumospirura rodentium* (Israel) |
| **13. Family:** Rhamnaceae  *Trichocephalus muris* (Egypt) |
| **14. Family:** Scarabaeidae  *Trichocephala* spp. (Iran) |
| **15. Family:** Spirocercidae  *Mastophorus muris* (Egypt, Iran), *Physocephalus sexalatus* (Iran) |
| **16. Family:** Spiruridae  *Protospirura marsupialis* (Egypt), *Protospirura muricola* (Egypt), *Protospirura muris* (Egypt), *Protospirura seurat* (Iran) |
| **17. Family:** Spiruroidea  *Streptopharagus kuntzi* (Egypt, Iran, Israel), *Streptopharagus numidicus* (Egypt) |
| **18. Family:** Strongylidae  *Strongyloides papillosus* (Egypt), *Strongyloides ratti* (Egypt, Turkey), *Strongyloides* spp. (Egypt, Iran, Palestine) |
| **19. Family:** Thelaziidae  *Gongylonema aegypti* (Egypt), *Gongylonema longispiculum* (Israel), *Gongylonema monigi* (Iran), *Gongylonema neoplasticum* (Iran), *Gongylonema pulchrum* (Iran), *Gongylonema* sp. (Iran, Israel), *Rictularia ratti* (Iran), *Rictularia* spp. (Iran) |
| **20. Family:** Trichinellidae  *Trichinella* sp. (Egypt) |
| **21. Family:** Trichosomoididae  *Trichosomoides crassicauda* (Iran, Israel), *Trichostrongylus* spp. (Iran) |
| **22. Family:** Trichuridae  *Capillaria annulosa* (Iran), *Capillaria hepatica* (Egypt, Iran, Turkey), *Capillaria* sp. (Iran), *Trichuris mofidii* (Iran), *Trichuris muris* (Egypt, Iran, Israel), *Trichuris rhombomidis* (Iran), *Trichuris* spp. (Egypt, Iran, Iraq, Turkey), *Trichuris trichiura* (Iran) |
| **23. Family:** Trychostrongylidae  *Heligmosomoides polygyrus* (Iran), *Heligmosomoides skrjabini* (Iran) |

**Table S3d** Prevailing trematodes in rodents of the Middle East

| **1. Family:** Brachylaimidae  *Scaphiostomum* sp. (Israel) |
| --- |
| **2. Family:** Cyathocotylidae  *Mesostephanus aegyptiacus, Mesostephanus rodentium, Mesostephanus* spp. (Egypt) |
| **3. Family:** Didymozoidae  *Coelomotrema aegyptiaca* (Egypt) |
| **4. Family:** Echinochasmidae  *Echinochasmus* sp. (Egypt) |
| **5. Family:** Echinostomatidae  *Echinoparyphium recurvatum, Echinostoma callawayensis, Echinostoma liei, Echoinostoma* sp. (Egypt) |
| **6. Family:** Fasciolidae  *Fasciola hepatica* (Egypt), *Fasciola* spp. (Egypt, Saudi Arabia) |
| **7. Family:** Heterophyidae  *Haplorchis pumilio, Haplorchis yokogawai, Heterophyes heterophyes, Pygidiopsis genata, Stictodora tridactyla* (Egypt) |
| **8. Family:** Lecithodendriidae  *Prosthodendrium ascidia, Prosthodendrium* spp. (Egypt) |
| **9. Family:** Notocotylidae  *Notocotylus neyrai* (Iran) |
| **10. Family:** Plagiorchiidae  *Plagiorchis muris* (Iran), *Plagiorchis* sp. (Egypt) |
| **11. Family:** Schistosomatidae  *Schistosoma mansoni* (Egypt), *Schistosoma* spp. (Egypt) |
